# Organizational readiness and implementation fidelity of an early childhood education and care-specific physical activity policy intervention: findings from the Play Active trial

**DOI:** 10.1093/pubmed/fdad221

**Published:** 2023-11-22

**Authors:** Elizabeth J Wenden, Charley A Budgeon, Natasha L Pearce, Hayley E Christian

**Affiliations:** Telethon Kids Institute, University of Western Australia, Crawley, WA, Australia; School of Population and Global Health, University of Western Australia, Crawley, WA, Australia; School of Population and Global Health, University of Western Australia, Crawley, WA, Australia; Telethon Kids Institute, University of Western Australia, Crawley, WA, Australia; School of Population and Global Health, University of Western Australia, Crawley, WA, Australia; Telethon Kids Institute, University of Western Australia, Crawley, WA, Australia; School of Population and Global Health, University of Western Australia, Crawley, WA, Australia

**Keywords:** childcare, early childhood, health promotion, implementation, physical activity, policy

## Abstract

**Background:**

Many children do not accumulate sufficient physical activity for good health and development at early childhood education and care (ECEC). This study examined the association between ECEC organizational readiness and implementation fidelity of an ECEC-specific physical activity policy intervention.

**Methods:**

Play Active aimed to improve the ECEC educator’s physical activity practices. We investigated the implementation of Play Active using a Type 1 hybrid study (January 2021–March 2022). Associations between organizational readiness factors and service-level implementation fidelity were examined using linear regressions. Fidelity data were collected from project records, educator surveys and website analytics.

**Results:**

ECEC services with higher levels of organizational commitment and capacity at pre-implementation reported higher fidelity scores compared to services with lower organizational commitment and capacity (all *P*s < 0.05). Similarly, services who perceived intervention acceptability and appropriateness at pre-implementation to be high had higher fidelity scores (*P* < 0.05). Perceived feasibility and organizational efficacy of Play Active were associated with higher but nonsignificant fidelity scores.

**Conclusions:**

Results indicate that organizational readiness factors may influence the implementation of ECEC-specific physical activity policy interventions. Therefore, strategies to improve organizational readiness should be developed and tested. These findings warrant confirmation in the ECEC and other settings and with other health behavior interventions.

## Background

Internationally, less than a quarter of young children 0–5 years of age, meet national and international 24-Hour Movement Guidelines for the Early Years.^1–6^ For physical activity and sedentary behaviors, these Guidelines^7–10^ state that, each day, children aged 2–5 years should accumulate ≥180 minutes of total physical activity, including ≥60 minutes of moderate to vigorous physical activity for 3–5-year-olds, and ≤60 minutes of sedentary/screen time. For infants aged 0–1 years, the recommendations are being physically active daily, including ≥30 minutes of tummy time, ≤ 60 minutes sedentary time and no screen time.^7–10^ A lack of sufficient physical activity and high levels of sedentary time in young children can lead to an increased risk of high blood pressure, insulin resistance, musculoskeletal problems, obesity, bullying and being socially isolated.^7^^,^^9^

Early childhood education and care (ECEC) is an ideal setting for interventions aimed at increasing young children’s physical activity, with between 50 and 90% of 0–5-year-old children attending out-of-home care globally.^11^^,^^12^ However, many young children do not accumulate sufficient physical activity for good health and development while at ECEC.^12^ Although increased levels of physical activity have been reported in some ECEC-based interventions, these results are often marginal and fade quickly.^12^^,^^13^ The potential for positive longer-term impact on physical activity practices through better organizational support and implementation strategies has yet to be explored.

ECEC educators are pivotal in shaping young children’s behaviors.^13^ However, competing educator work priorities can be a barrier when implementing changes in policy or practice. Few studies have focused on the key contextual, organizational and individual barriers to implementation of physical activity interventions in ECEC.^12^^,^^14^ However, a lack of training, negative educator mindset, lack of resources, parent and educator risk aversion, dissent about physical activity as a learning experience,^15^ limited technological capacity and staff turnover^16^ have been reported as implementation barriers to a new ECEC-specific physical activity policy.^15^ While addressing these barriers is an important step, there is no guarantee that ECEC-specific physical activity interventions will be translated into practice by educators if services are not adequately prepared and ‘ready’ to implement.^15^

Currently, organizational readiness is not commonly measured for any type of intervention in the ECEC setting.^17^ Organizational readiness is a multifaceted concept, evident across all levels of an organization and comprises notions of shared resolve, motivation and collective capacity to undertake practice change.^18^ Organizational readiness typically measures factors known to be associated with the early adoption and implementation of a new intervention, such as perceived levels of organizational commitment, capacity and efficacy on the basis that the proposed intervention is better than the status quo.^17–22^ Pre-implementation knowledge about the perceived acceptability, appropriateness and feasibility of an intervention by an organization is also recognized as useful for the early mitigation of implementation barriers.^23^^,^^24^ While often used as outcome measures of implementation quality,^23^ we propose that ‘organizational readiness’ is a concept that should include the pre-implementation knowledge of intervention acceptability, appropriateness and feasibility. Acceptability defines how agreeable an intervention is perceived to be, whereas appropriateness denotes the perceived intervention relevance.^24^ Feasibility is the perceived extent to which the intervention can be implemented.^24^ In effect, perceived acceptability, appropriateness and feasibility are organizational characteristics that indicate the compatibility of an intervention with a particular organizational setting.^25^ This recognition and a belief that a positive benefit will result are key in determining the fit of the intervention to the organization.^23–25^

Organizational readiness influences intervention implementation success.^26^^,^^27^ In the ECEC setting, these factors that can help with ensuring educators are ‘on the same page’ as their service director/leader,^15^^,^^28^ can help overcome implementation barriers by increasing intervention knowledge, promote educator participation and support adoption of new practices by helping educators feel confident in their abilities to adapt to new policies and procedures.^29^ Given this, further knowledge of organizational readiness factors as tools for supporting the implementation of ECEC-specific physical activity interventions requires investigation.

Organizational readiness is critical to implementation fidelity.^30^^,^^31^ Implementation fidelity explains ‘what works’ in effectiveness trials,^32^^,^^33^ informs study replication,^33^ supports practical application^32^^,^^33^ and identifies protocol variations.^33^ The measurement and reporting of implementation fidelity can provide information about whether implementation strategies are sufficient and if additional or different strategies are needed.^34^ A recent review found that the level of implementation of ECEC-specific policies and practices were low and implementation strategies were insufficient to change these outcomes.^12^ Measuring implementation fidelity, therefore, is necessary for understanding the success of ECEC-specific physical activity interventions. Further research is needed to understand the specific organizational readiness factors that influence the implementation fidelity of health interventions in this setting. The aim of this study, therefore, was to examine the associations between ECEC organizational readiness factors and the implementation fidelity of an ECEC-specific physical behavior policy intervention—Play Active.

## Methods

A pragmatic cluster randomized trial evaluated the effectiveness of the Play Active physical activity policy intervention to improve ECEC educators physical activity-related practices.^35^ The primary outcome of Play Active was reported change in educator physical activity-related practices and is described in detail in the published protocol.^35^ This study measured the implementation fidelity of Play Active as a secondary outcome using a Type 1 Hybrid effectiveness-implementation design.^36^ This study design maintains the focus on intervention effectiveness outcomes while allowing exploration of the intervention’s implementation.^36^

Play Active is an evidence-informed ECEC-specific physical activity policy template with nine physical activity and sedentary time recommendations for young children in care.^35^ The policy intervention is underpinned by six implementation strategies (implementation intervention): (i) personalization of the policy, (ii) policy review and approval, (iii) resource guide mapped to policy practices, (iv) Energetic Play Assessment Tool (EPAT) to monitor young children’s physical activity levels, (v) online professional development and (vi) technical assistance for implementation support.^35^ After tailoring the Play Active policy template to suit service needs, directors completed a pre-implementation survey. A 3–5-month period was provided for the implementation of Play Active, after which service directors completed a post-implementation (follow up) survey. Ethics approval was provided by the University of Western Australian Human Ethics Research Committee (RA/4/20/6120).

### Participants and setting

Long day ECEC services caring for children aged 0–5 years with a minimum 20 enrolled children and located in the Perth, Australia metropolitan area were eligible to take part. Services were invited to complete an expression of interest (EOI) to participate. Information and consent packs were provided to each service. Consenting services were randomly allocated to either the intervention group (*n* = 40) or the wait-listed control group (*n* = 40). Only intervention services participated in the current study.

### Data collection and measures

Service directors completed a survey in the period after tailoring their physical activity policy and prior to commencing implementation (pre-implementation period: January to June 2021). The post-intervention survey was collected from September 2021 to March 2022 (post-implementation period). In this study, data were either collected at the service-level or summarized to the service-level for consistency.

#### Demographic measures

Service-level demographics were collected at pre-implementation. Directors' level of education was coded into two categories (lower: secondary school/certificate/diploma versus higher: university degree). Service-level data were sourced from the Australian Children’s Education and Care Quality Authority (ACECQA) website^37^ and included service ratings for the ‘Quality Rating 2: Children’s health and safety’ element: ‘Each child’s health and physical activity is supported and promoted’ (ratings: not assessed, working toward, meeting and exceeding) and number of approved places to determine service size (small/medium ≤57 children, large = 58–74 children and very large >75 children). Service suburb postcode was used as a proxy for socio-economic status (SES) (low, middle and high) and derived from the Australian Bureau of Statistics’ Socio-Economic Indexes for Areas (SEIFA).^38^

#### Organizational readiness measures

The 12-item Organizational Readiness for Implementing Change (ORIC)^39^ measured ECEC service change commitment and change efficacy at pre-implementation (see Supplementary Table 1: Organizational Readiness Measures). The ORIC has been shown to be reliable (α = 0.85–0.94)^39–41^ and suitable for use at the organizational level.^39^ Organizational capacity was measured at pre-implementation by the 5-item organizational capacity scale from the Program Sustainability Assessment Tool (PSAT) (see Supplementary Table 1).^42^ The PSAT tool is reliable, with the organizational capacity scale reporting α = 0.87 for internal consistency.^42^

The Acceptability of Intervention Measure (AIM), Intervention Appropriateness Measure (IAM) and Feasibility of Intervention Measure (FIM) scales, developed by Weiner and colleagues,^24^ measured directors’ perceptions of the acceptability, appropriateness and feasibility of Play Active at pre-implementation (Supplementary Table 1). The AIM, IAM and FIM 4-item scales are structurally valid (AIM α = 0.85, IAM α = 0.91, FIM α = 0.89), reliable (test–retest: AIM α = 0.83, IAM α = 0.87, FIM α = 0.88) and designed to be customisable.^24^

Due to > 95% of responses falling into the two highest of the five response for each of organizational commitment, efficacy, capacity and intervention acceptability, appropriateness and feasibility scales, responses were recoded into two categories as shown in Table 1.

**Table 1:** Recoding of organizational readiness response variables for analysis

Measure	Original scoring	Recoded for analysis
Adapted ORIC change efficacy and commitment scales^39^	5-pt Likert: (1) Disagree (2) Somewhat disagree (3) Neither agree nor disagree (4) Somewhat agree (5) Agree	(1) Some or neutral efficacy, some or neutral commitment (1–4, recoded to 1) (2) Efficacious, committed (5, recoded to 2)
Adapted PSAT organizational capacity scale^42^	5-pt Likert: (1) No extent (2) Little extent (3) Some extent (4) Great extent (5) Very great extent	(1) Some or neutral commitment (1–4, recoded to 1) (2) Very great extent (5, recoded to 2)
Customized AIM, IAM and FIM measures^24^	5-pt Likert: (1) Disagree (2) Somewhat disagree (3) Neither agree nor disagree (4) Somewhat agree (5) Agree	(1) Somewhat acceptable (AIM), somewhat appropriate (IAM) or somewhat feasible (SIM) (1–4, recoded to 1) (2) Acceptable (AIM), appropriate (IAM) or feasible (FIM) (5, recoded to 2)

#### Fidelity measure

**Fig. 1:**
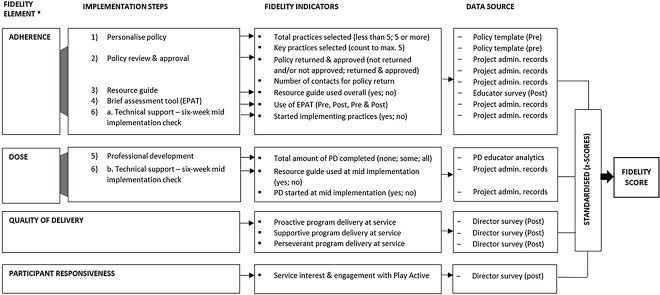
Development of fidelity measure. Program differentiation not measured. PD, professional development; Pre, pre-implementation data collection; Post, post-implementation data collection.

Informed by the work of Proctor *et al*.^23^ a fidelity measure was constructed to measure overall implementation fidelity, adherence, dose, quality of delivery and participant responsiveness scores of Play Active at post-implementation (Fig. 1). These were based on fidelity indictors set out in the Play Active protocol (e.g. personalize policy: services select at least five from 25 practices within the physical activity policy template to focus on initially).^35^ Sources for fidelity indicator data included web analytics, selected educator evaluation survey questions and project administration records. The components of the fidelity measure items were converted to *z*-scores to produce individual scores for adherence, dose, quality of delivery and participant responsiveness and then summed to produce an overall fidelity score (Supplementary Table S2).

### Data analysis

Using SPSS v.28,^43^ descriptive statistics were calculated for all variables including frequencies and percentages. Independent *t*-tests were used to analyze unadjusted associations that compared the overall fidelity score between those with, e.g. some or neutral commitment against those who were committed, for each independent variable (organizational commitment, efficacy and capacity; acceptability, appropriateness and feasibility). Subsequently, linear regression was used to model the six organizational readiness variables separately in relation to the overall fidelity score. All models were adjusted for ECEC service socio-demographic characteristics including service size, SES, quality rating for children’s health and safety and director’s education level. Model assumptions were thoroughly checked and confirmed to ensure the validity of the analyses.

## Results

Most ECEC intervention services (73%) were large to very large with 58 or more approved places for children (Table 2). Around half of services (48%) were in high SES areas and just over half (55%) were rated as ‘meeting the national quality standard’ for ‘Quality Rating 2: Children’s health and safety’, which includes promoting children’s physical activity.^44^ The majority of ECEC service directors (80%) did not hold tertiary education qualifications.

**Table 2:** ECEC service sample characteristics

Sample characteristics (*n* = 36)		*n* (%)
Service size	Small/medium—0–57 children	11 (27.5)
	Large—58–74 children	13 (32.5)
	Very large—>75 children	16 (40.0)
Service socio-economic status	Low	13 (32.5)
	Medium	8 (20.0)
	High	19 (47.5)
Quality rating assessment:	Working toward quality standard	8 (20.0)
Children’s health and safety	Meeting quality standards	24 (60.0)
	Exceeding quality standards	8 (20.0)
Director’s educational level	Secondary school/certificate/diploma or lower	32 (80.0)
	University degree or higher	8 (20.0)
ORIC commitment scale^a^^,^^39^	Some commitment or neutral	14 (38.9)
	Committed	22 (61.1)
ORIC efficacy scale^a^^,^^39^	Some efficacy or neutral	10 (27.8)
	Efficacious	26 (72.2)
Organizational capacity scale (PSAT)^a^^,^^42^	Some to great extent	25 (69.4)
	Very great extent	11 (30.6)
AIM^a^^,^^24^	Somewhat acceptable or neutral	15 (41.7)
	Acceptable	21 (58.3)
IAM^a^^,^^24^	Somewhat appropriate or neutral	9 (25.7)
	Appropriate	26 (74.3)
FIM^a^^,^^24^	Somewhat feasible or neutral	9 (25.7)
	Feasible	16 (74.3)

^a^Missing = 4.

^b^Mean ± standard deviation.

Most directors reported their ECEC service had commitment (61.1%) and efficacy (72.2%) in place to implement Play Active whereas about one-third (30.6%) reported having the organizational capacity to change to a ‘very great’ extent. While in pre-implementation, services were almost equally split on the level of acceptability of Play Active (somewhat acceptable/neutral 41.7% versus acceptable 58.3%). Most reported that the intervention was appropriate and/or feasible (both 74.3%).

The overall fidelity mean *z*-score for ECEC services was 1.7 ± 5.0 (possible range: −7.97 to 12.33) at the 3–5-month follow-up. The fidelity components of adherence, dose, quality of delivery and participant responsiveness were examined by service demographic variables (Supplementary Fig. 1a and b). More ECEC services met the dose criteria in the ‘exceeding’ quality rating for Children’s Health and Safety (67%) compared with those who had a ‘meeting’ quality rating (25%) or were `working toward'/`not assessed' (38%) (*P* = 0.027). More small/medium (91%) or large ECEC services (92%) met the participant responsiveness criteria than very large services (63%) (*P* < 0.048). No other significant socio-demographic differences were found.

Unadjusted associations between the six organizational readiness baseline variables and the post-implementation fidelity score reported significant results for all except feasibility (Supplementary Table 3).

**Table 3:** Multivariable linear regression of Play Active fidelity score and baseline organizational readiness

		Fidelity score (*n* = 36)						
		Model 1		Model 2	Model 3	Model 4	Model 5	Model 6
		MD (95% CI)		MD (95% CI)	MD (95% CI)	MD (95% CI)	MD (95% CI)	MD (95% CI)
Organizational commitment:								
Committed versus some/neutral commitment		4.96 (1.62, 8.29)^*^		—	—	—	—	—
						
Organizational efficacy:								
Efficacious versus some/neutral efficacy		—		3.22 (−1.00, 7.43)	—	—	—	—
				
Organizational capacity:								
Very great extent versus some/great extent		—		—	4.11 (0.78, 7.43)^*^	—	—	—
				
Acceptability of Intervention:								
Acceptable versus somewhat acceptable/neutral		—		—	—	4.02 (0.63, 7.40)^*^	—	—
						
Appropriateness of Intervention:								
Appropriate versus somewhat appropriate/neutral		—		—	—	—	5.13 (1.32, 8.93)^*^	—
						
Feasibility of Intervention:								
Feasible versus somewhat feasible/neutral		—		—	—	—	—	1.39 (−2.38, 5.16)
						
Adjustment variables:								
Service size^a^	Small/medium	Ref		Ref	Ref	Ref	Ref	Ref
	Large	−2.70 (−6.71, 1.31)		−3.20 (−7.76, 1.37)	−4.30 (−8.30, −0.31)^*^	−3.23 (−7.39, 0.92)	−4.17 (−8.17, −0.17)^*^	−4.35 (−8.69, −0.01)^*^
Very large		−2.85 (−6.62, 0.91)		−2.67 (−6.90, 1.56)	−2.67 (−6.61, 1.27)	−1.92 (−6.01, 2.17)	−3.84 (−7.76, 0.09)	−2.79 (−7.15, 1.56)
						
Service SES	Low	Ref		Ref	Ref	Ref	Ref	Ref
Middle		0.44 (−3.96, 4.84)		1.74 (−3.07, 6.55)	3.44 (−1.31, 8.18)	1.68 (−2.87, 6.22)	−0.20 (−3.90, 3.49)	1.65 (−3.23, 6.53)
High		−0.47 (−3.83, 2.88)		−0.35 (−4.43, 3.72)	−1.10 (−4.51, 2.32)	−0.61 (−4.16, 2.93)	2.96 (−1.66, 7.60)	−1.11 (−4.86, 2.64)
						
Quality rating 2^b^	Not assessed	Ref		Ref	Ref	Ref	Ref	Ref
Meeting rating		1.83 (−2.09, 5.75)		1.65 (−2.71, 6.00)	2.99 (−1.21, 7.19)	2.94 (−1.29, 7.18)	2.54 (−1.51, 6.58)	2.24 (−2.22, 6.71)
Exceeding rating		3.84 (−1.41, 9.09)		5.07 (−0.68, 10.82)	7.50 (2.16, 12.83)^*^	5.42 (0.12, 10.72)^*^	4.56 (−0.68, 9.81)	5.99 (0.26, 11.73)*
						
Director’s education level	Secondary school certificate/diploma or less	Ref		Ref	Ref	Ref	Ref	Ref
University degree or higher		4.43 (0.81, 8.03)^*^		3.41 (−0.55, 7.38)	2.68 (−1.10, 6.47)	2.50 (−1.35, 6.35)	3.37 (−0.28, 7.02)	4.41 (0.12, 8.70)^*^

— denotes not included in analysis

^*^ *P* < 0.05

^a^Small/medium, 0–57 children; large, 58–74 children; very large, 75 children.

^b^Quality rating 2, Children’s Health and Safety.

### Adjusted associations between organizational readiness and Play Active implementation fidelity

After adjusting for service-level characteristics, ECEC services who were fully ‘committed’ to implementing Play Active reported significantly higher fidelity than services with ‘some or neutral commitment’ (MD 4.96; 95% CI 1.62–8.29; *P* = 0.005) (Table 3). Similarly, ECEC services who had organizational capacity to a ‘very great extent’ reported significantly higher fidelity scores than services with less than to a ‘very great extent’ (MD 4.11; 95% CI 0.78, 7.43; *P* = 0.017). No significant results were found between organizational efficacy and fidelity; however, higher fidelity scores were seen in services reporting Play Active as ‘efficacious’ over those reporting ‘some or neutral efficacy’.

ECEC services who reported Play Active was acceptable or appropriate at pre-implementation had significantly higher fidelity scores compared to services with lower levels of perceived acceptability or appropriateness (MD 4.02, 95% CI 0.63–7.40, *P* = 0.022, and MD 5.13, 95% CI: 1.32–8.93, *P* = 0.010, respectively) (Table 3). The perceived feasibility of Play Active was associated with higher but non-significant fidelity scores.

## Discussion

### Main findings of the study

This study examined the association between ECEC service organizational readiness factors and the implementation fidelity of Play Active. Higher levels of Play Active implementation fidelity were associated with higher levels of organizational commitment and organizational capacity for change. Our results also showed that ECEC services with higher implementation fidelity of Play Active had higher levels of pre-implementation intervention acceptability and appropriateness. Overall, implementation fidelity to Play Active was positively associated with factors of organizational readiness.

### What is already known on this topic

Evidence from the health and education sectors reports high organizational readiness enables better implementation of health interventions.^18–20^^,^^45^^,^^46^ More recently, Metz and colleagues^47^ used an ECEC-specific lens with implementation science concepts to suggest that higher levels of organizational readiness may point to the development of an ‘hospitable environment’^47^ for successful implementation of, and fidelity to, evidence-based interventions in the ECEC setting. In addition, previous work has examined the use of strategies to increase acceptability and appropriateness of childhood obesity interventions.^23^^,^^48^ Improving intervention ‘fit’ at the service-level can be facilitated by strategies such as pre-intervention training and planning to engage and incorporate expertise of potential end-users (i.e. ECEC staff). This promotes ownership of practice change and can ensure implementation aligns with the setting’s needs, priorities and available resources.^15^^,^^28^^,^^29^^,^^45^ The use of such strategies is important given an increasing focus on ‘designing for dissemination and implementation’^24^ and requirements for the substantial time and costs associated with intervention development to represent value for money.^45^

### What this study adds

The purposeful creation of an hospitable environment through organizational readiness strategies (e.g. two-way communication, quality improvement processes and time for professional development) in ECEC services may be necessary to support end-user engagement with implementation,^49^ promote intervention fidelity^46^ and to support ownership of the resultant practice changes.^45^ Play Active’s use of such strategies (e.g. educator engagement in Play Active development) may have facilitated the high levels of commitment, capacity, acceptability and appropriateness reported. Practical strategies that may increase organizational readiness in ECEC include the provision of sufficient educators to cover for those undertaking professional development, more/new play equipment, methods in place to track changes in children’s physical activity and early engagement of parents/carers and other stakeholders,^47^ talking with educators regularly (informally and formally) about the new policy they will implement and getting educator input as to their support needs for implementation success.^15^ Overall, our findings provide support for the relationships between organizational readiness factors and implementation fidelity. However, further research is required to clarify these relationships and develop strategies to improve organizational readiness prior to implementation.

These results may have been influenced by the timing of the Play Active intervention. Play Active was implemented during the Covid-19 pandemic, which involved various restrictions (e.g. fewer children in care due to mandatory lockdown). Higher levels of staff turnover, increased sick leave, isolation restrictions, longer work hours, stress and burnout among ECEC staff resulted in less time for planning or implementing^50^ a new physical activity policy. Despite this, our results indicate organizational readiness is key to implementation fidelity for ECEC-specific physical activity policy, however, further research is needed to confirm these findings post-Covid-19 restrictions.

### Limitations of the study

The Play Active trial was subject to limitations that impacted the current study, including: a relatively short implementation period, self-selection of the ECEC sample through the EOI process and ECEC workforce challenges exacerbated by Covid-19 restrictions. Participating ECEC services were metropolitan only, impacting the generalizability of our results for implementing Play Active in non-metropolitan areas. Minimal variation across some survey item responses resulted in response scales being dichotomized for data analysis. In addition, the measures used were either from other settings or were developed for the current study and therefore were not validated in ECEC settings. ECEC service visits were forbidden during the implementation period (Covid-19) and relied on service director self-reports rather than objective observations of the implementation of Play Active. Finally, as only long daycare services participated in the main study, these results may not be applicable to other ECEC types (e.g. family day care and out of school care).

## Conclusions

This study explored the relationships between organizational readiness and implementation fidelity of the ECEC-based Play Active physical activity policy intervention. Our results demonstrated that increases in Play Active implementation fidelity were significantly associated with higher levels of organizational readiness factors. These results are important for informing the future implementation and sustainability of physical activity interventions in the ECEC setting as many rarely make significant or lasting increases in children’s activity levels. To support successful implementation and the subsequent positive impact on child physical activity levels and health, further research should be undertaken to determine how to develop and incorporate organizational readiness strategies into future ECEC-specific physical activity policy interventions.

## Supplementary Material

Supplementary_materials_fdad221

## Data Availability

The data underlying this article may be shared on reasonable request to the corresponding author.

## Notes

**Elizabeth J. Wenden,** PhD Candidate

**Charley A. Budgeon,** Lecturer in Public Health/Biostatistician

**Natasha L. Pearce,** Senior Research Fellow

**Hayley E. Christian,** Senior Research Fellow and Head, Child Physical Activity, Health and Development

## References

[1] Rollo S, Antsygina O, Tremblay MS. The whole day matters: understanding 24-hour movement guideline adherence and relationships with health indicators across the lifespan. J Sport Health Sci 2020;9(6):493–510. 32711156 10.1016/j.jshs.2020.07.004 PMC7749249

[2] Hesketh KR, Lakshman R, van Sluijs EMF. Barriers and facilitators to young children’s physical activity and sedentary behaviour: a systematic review and synthesis of qualitative literature. Obes Rev 2017;18(9):987–1017. 28589678 10.1111/obr.12562 PMC5575514

[3] Christian H, Murray K, Trost SG et al. Meeting the Australian 24-Hour Movement Guidelines for the Early Years is associated with better social-emotional development in preschool boys. Prev Med Rep 2022;27:101770. 35321215 10.1016/j.pmedr.2022.101770 PMC8935500

[4] Feng J, Zheng C, Sit CH et al. Associations between meeting 24-hour movement guidelines and health in the early years: a systematic review and meta-analysis. J Sports Sci 2021;39(22):2545–57. 34176439 10.1080/02640414.2021.1945183

[5] McWilliams C, Ball S, Benjamin S et al. Best-practice guidelines for physical activity at child care. Pediatrics 2009;124(6):1650–9. 19917582 10.1542/peds.2009-0952

[6] Sisson SB, Campbell JE, May KB et al. Assessment of food, nutrition, and physical activity practices in Oklahoma child-care centers. J Acad Nutr Diet 2012;112(8):1230–40. 22818731 10.1016/j.jand.2012.05.009

[7] Australian Government Department of Health . Australian 24-Hour Movement Guidelines for the Early Years (Birth to 5 years): An integration of physical activity, sedentary behaviour, and sleep. Commonwealth of Australia. https://www.health.gov.au/internet/main/publishing.nsf/Content/npra-0-5yrs-brochure (26 May 2019, date last accessed).

[8] Tremblay MS, Chaput J-P, Adamo KB et al. Canadian 24-Hour Movement Guidelines for the Early Years (0–4 years): an integration of physical activity, sedentary behaviour, and sleep. BMC Public Health 2017;17(5):874. 29219102 10.1186/s12889-017-4859-6 PMC5773896

[9] World Health Organization . Guidelines on Physical Activity, Sedentary Behaviour and Sleep for Children under 5 Years of Age. World Health Organisation. https://apps.who.int/iris/handle/10665/311664 (30 May 2019, date last accessed). 31091057

[10] Chief Medical Officers UK . UK Chief Medical Officers’ Physical Activity Guidelines. Department of Health and Social Care Llwodraeth Cymru Welsh Government, Department of Health Northern Ireland and the Scottish Government, Department of Health and Social Care, England. https://assets.publishing.service.gov.uk/government/uploads/system/uploads/attachment_data/file/832868/uk-chief-medical-officers-physical-activity-guidelines.pdf (7 July 2021, date last accessed).

[11] OECD Social Policy Division - Directorate of Employment Labour and Social Affairs . Enrolment in Childcare and Pre-School (Public policies for Families and Children). www.oecd.org/els/soc/PF3_2_Enrolment_childcare_preschool.pdf (11 May 2021, date last accessed).

[12] Wolfenden L, Barnes C, Jones J et al. Strategies to improve the implementation of healthy eating, physical activity and obesity prevention policies, practices or programmes within childcare services. Cochrane Database Syst Rev 2020;2020:CD011779. 10.1002/14651858.CD011779.pub3 PMC7008062 32036618

[13] Hesketh KR, O'Malley C, Paes VM et al. Determinants of change in physical activity in children 0–6 years of age: A systematic review of quantitative literature. Sports Med 2017;47(7):1349–74. 27988875 10.1007/s40279-016-0656-0 PMC5488114

[14] Jackson JK, Jones J, Nguyen H et al. Obesity prevention within the early childhood education and care setting: a systematic review of dietary behavior and physical activity policies and guidelines in high income countries. Int J Environ Res Public Health 2021;18(2):838. 33478165 10.3390/ijerph18020838 PMC7835808

[15] Wenden EJ, Pearce N, George P et al. Educators’ barriers and facilitators to physical activity policy implementation in the childcare setting: qualitative findings from the Play Active project. Am J Health Promot 2022;36(8):1326–34. 35612922 10.1177/08901171221105052

[16] Kenney EL, Mozaffarian RS, Ji W et al. Moving from policy to practice for early childhood obesity prevention: a nationwide evaluation of state implementation strategies in childcare. Int J Environ Res Public Health 2022;19(16):10304. 36011939 10.3390/ijerph191610304 PMC9408404

[17] Halle T, Partika A, Nagle K. Measuring Readiness for Change in Early Care and Education, OPRE Report #201963. Office of Planning, Research and Evaluation, Administration for Children and Families, U.S. Department of Health and Human Services. https://www.acf.hhs.gov/opre (10 March 2023, date last accessed).

[18] Weiner BJ . A theory of organizational readiness for change. Implement Sci 2009;4(1):67. 19840381 10.1186/1748-5908-4-67 PMC2770024

[19] Alolabi YA, Ayupp K, Dwaikat MA. Issues and implications of readiness to change. Adm Sci 2021;11(4):140.

[20] Arthur K, Christofides N, Nelson G. Educators’ perceptions of organisational readiness for implementation of a pre-adolescent transdisciplinary school health intervention for inter-generational outcomes. PloS One 2020;15(1):e0227519. 31914148 10.1371/journal.pone.0227519 PMC6948754

[21] Scaccia JP, Cook BS, Lamont A et al. A practical implementation science heuristic for organisational readiness: R = mc^2^. J Community Psychol 2015;43(4):484–501. 26668443 10.1002/jcop.21698 PMC4676714

[22] Sharma SV, Upadhyaya M, Schober DJ et al. A conceptual framework for organizational readiness to implement nutrition and physical activity programs in early childhood education settings. Prev Chronic Dis 2014;11:E190. 25357258 10.5888/pcd11.140166 PMC4215571

[23] Proctor E, Silmere H, Raghavan R et al. Outcomes for implementation research: conceptual distinctions, measurement challenges, and research agenda. Adm Policy Ment Health 2011;38(2):65–76. 20957426 10.1007/s10488-010-0319-7 PMC3068522

[24] Weiner BJ, Lewis CC, Stanick C et al. Psychometric assessment of three newly developed implementation outcome measures. Implement Sci 2017;12(1):108–8. 28851459 10.1186/s13012-017-0635-3 PMC5576104

[25] Durlak JA, DuPre EP. Implementation matters: A review of research on the influence of implementation on program outcomes and the factors affecting implementation. Am J Community Psychol 2008;41(3–4):327–50. 18322790 10.1007/s10464-008-9165-0

[26] Allen P, Pilar M, Walsh-Bailey C et al. Quantitative measures of health policy implementation determinants and outcomes: a systematic review. Implement Sci 2020;15(1):47. 32560661 10.1186/s13012-020-01007-w PMC7304175

[27] Serhal E, Arena A, Sockalingam S et al. Adapting the consolidated framework for implementation research to create organizational readiness and implementation tools for project ECHO. J Contin Educ Health Prof 2018;38(2):145–51. 29505486 10.1097/CEH.0000000000000195 PMC5999379

[28] Heikka J, Pitkäniemi H, Kettukangas T et al. Distributed pedagogical leadership and teacher leadership in early childhood education contexts. Int J Leadersh Educ 2019;24:333–48.

[29] Doyle O, Logue C, McNamara KA. Readiness to implement a national quality framework: Evidence from Irish early childhood care and education centres. Child Care Pract 2011;17(2):163–84.

[30] Locke J, Kang-Yi C, Frederick L et al. Individual and organizational characteristics predicting intervention use for children with autism in schools. Autism 2020;24(5):1152–63. 31867987 10.1177/1362361319895923 PMC7308214

[31] Vax S, Farkas M, Russinova Z et al. Enhancing organizational readiness for implementation: constructing a typology of readiness-development strategies using a modified Delphi process. Implement Sci 2021;16(1):61. 34112191 10.1186/s13012-021-01132-0 PMC8194182

[32] Gage N, Macsuga A, Detrich R, States J. Fidelity of Implementation in Educational Research and Practice. Oakland, USA: The Wing Institute. https://www.winginstitute.org/systems-program-fidelity (29 June 2023, date last accessed).

[33] Sanchez-Flack JC, Herman A, Buscemi J et al. A systematic review of the implementation of obesity prevention interventions in early childcare and education settings using the RE-AIM framework. Transl Behav Med 2020;10(5):1168–76. 33044537 10.1093/tbm/ibz179 PMC7549410

[34] Lemire C, Dionne C, Rousseau M. Fidelity of implementation of activity-based intervention in daycare. Early Child Dev Care 2022;192(9):1368–83.

[35] Nathan A, Adams E, Trost S et al. Evaluating the effectiveness of the Play Active policy intervention and implementation support in early childhood education and care: a pragmatic cluster randomised trial protocol. BMC Public Health 2022;22(1):306. 35164729 10.1186/s12889-022-12729-5 PMC8842565

[36] Curran GM, Bauer M, Mittman B et al. Effectiveness-implementation hybrid designs: combining elements of clinical effectiveness and implementation research to enhance public health impact. Med Care 2012;50(3):217–26. 22310560 10.1097/MLR.0b013e3182408812 PMC3731143

[37] Australian Children’s Education & Care Quality Authority . Australian Children's Education & Care Quality Authority. ACECQA. https://www.acecqa.gov.au/ (15 June 2022, date last accessed).

[38] Australian Bureau of Statistics . Socio-Economic Indexes for Areas (SEIFA). ABS. https://www.abs.gov.au/websitedbs/censushome.nsf/home/seifa (10 June 2022, date last accessed).

[39] Shea CM, Jacobs SR, Esserman DA et al. Organizational readiness for implementing change: a psychometric assessment of a new measure. Implement Sci 2014;9:7. 24410955 10.1186/1748-5908-9-7 PMC3904699

[40] Adelson P, Yates R, Fleet J-A et al. Measuring organizational readiness for implementing change (ORIC) in a new midwifery model of care in rural South Australia. BMC Health Serv Res 2021;21(1):368–8. 33879145 10.1186/s12913-021-06373-9 PMC8056551

[41] Ruest M, Léonard G, Thomas A et al. French cross-cultural adaptation of the Organizational Readiness for Implementing Change (ORIC). BMC Health Serv Res 2019;19(1):535. 31366390 10.1186/s12913-019-4361-1 PMC6668068

[42] Luke DA, Calhoun A, Robichaux CB et al. The Program Sustainability Assessment Tool: a new instrument for public health programs. Prev Chronic Dis 2014;11:130184. 24456645 10.5888/pcd11.130184 PMC3900326

[43] IBM SPSS Statistics for Windows, Version 28.0. Armonk, NY: IBM Corp, 2021.

[44] Australian Children’s Education & Care Quality Authority . Quality Area 2 – Children’s health and safety. ACECQA. https://www.acecqa.gov.au/nqf/national-quality-standard/quality-area-2-childrens-health-and-safety (11 July 2019, date last accessed).

[45] Leeman J, Rohweder C, Lee M et al. Aligning implementation science with improvement practice: a call to action. Implement Sci Commun 2021;2(1):99. 34496978 10.1186/s43058-021-00201-1 PMC8424169

[46] Bast LS, Andersen HB, Andersen A et al. School coordinators’ perceptions of organizational readiness is associated with implementation fidelity in a smoking prevention program: findings from the X:IT II study. Prev Sci 2021;22(3):312–23. 33404969 10.1007/s11121-020-01197-1 PMC8032573

[47] Metz A, Naoom SF, Halle T. et al. An Integrated Stage-Based Framework for Implementation of Early Childhood Programs and Systems (OPRE Research Brief OPRE 2015­). Washington, DC: Office of Planning, Research and Evaluation, Administration for Children and Families, U.S. Department of Health and Human Services. 2015.

[48] Gittelsohn J, Novotny R, Trude ACB et al. Challenges and lessons learned from multi-level multi-component interventions to prevent and reduce childhood obesity. Int J Environ Res Public Health 2019;16(1):30. 10.3390/ijerph16010030 PMC6339209 30586845

[49] Romero LM, Olaiya O, Hallum-Montes R et al. Efforts to increase implementation of evidence-based clinical practices to improve adolescent-friendly reproductive health services. J Adolesc Health 2017;60(3s):S30–s37. 28235433 10.1016/j.jadohealth.2016.07.017 PMC6650772

[50] Community Early Learning Australia, Community Child Care, Early Learning Association Australia . Investing in Our Future: Growing the Education and Care Workforce. CELA, CCC, ELAA. https://www.cela.org.au/CELA/Publications/Reports/Investing-in-our-Future-25-Nov-2021.pdf (11 October 2022, date last accessed).

