# Supplementary material for: Organizational readiness and implementation fidelity of an early childhood education and care-specific physical activity policy intervention: findings from the Play Active trial
Source: J Public Health (Oxf). 2023 Nov 22;46(1):158–67. doi: 10.1093/pubmed/fdad221 (PMC10901271; doi:10.1093/pubmed/fdad221)
Supplement: Supplementary_materials_fdad221 [file supplementary_materials_fdad221.zip › Supplementary_materials_fdad221/STROBE-checklist-v4-combined-PlosMedicine.docx]

STROBE Statement—checklist of items that should be included in reports of observational studies

|  | Item No. | Recommendation | Page  No. | Relevant text from manuscript |
| --- | --- | --- | --- | --- |
| **Title and abstract** | 1 | (*a*) Indicate the study’s design with a commonly used term in the title or the abstract | 1 | Play Active aimed to improve ECEC educator's physical activity practices. We investigated the implementation of Play Active using a Type 1 hybrid study (January 2021-March 2022). |
|  |  | (*b*) Provide in the abstract an informative and balanced summary of what was done and what was found | 1-2 | Associations between organisational readiness factors and service-level implementation fidelity was examined using linear regressions. Fidelity data were collected from project records, educator surveys and website analytics.  **Results:**  ECEC services with higher levels of organisational commitment and capacity at pre-implementation reported higher fidelity scores compared to services with lower organisational commitment and capacity (all p<0.05). Similarly, services who perceived intervention acceptability and appropriateness at pre-implementation to be high had higher fidelity scores (p<0.05). Perceived feasibility and organisational efficacy of Play Active were associated with higher but non-significant fidelity scores. |
| Introduction | | | |  |
| Background/rationale | 2 | Explain the scientific background and rationale for the investigation being reported | 3-4 | - Less than a quarter of young children meet national and international 24-Hour Movement Behaviour (physical activity, sedentary behaviour and sleep) Guidelines for the Early Years. - However, many young children do not accumulate sufficient physical activity for good health and development while at ECEC. - Although increased levels of physical activity have been reported in some ECEC-based interventions, these results are often marginal and fade quickly. - Currently, organisational readiness (commitment, efficacy, capacity) is not commonly measured for any type of intervention in the ECEC setting. Additionally, pre-implementation knowledge about the perceived acceptability, appropriateness and feasibility of an intervention is useful for mitigating potential problems with implementation. - Organisational readiness and intervention acceptability, appropriateness and feasibility are critical to implementation fidelity. - Measuring implementation fidelity, therefore, is necessary for understanding the success or otherwise of ECEC-specific physical activity interventions. |
| Objectives | 3 | State specific objectives, including any prespecified hypotheses | 4 | The aim of this study, therefore, was to examine the association between ECEC organisational readiness and intervention acceptability, appropriateness and feasibility, with the implementation fidelity of an ECEC-specific physical behaviour policy intervention – Play Active. |
| Methods | | | |  |
| Study design | 4 | Present key elements of study design early in the paper | 5 | The current study measured the implementation fidelity of Play Active as a secondary outcome using a Type 1 Hybrid effectiveness-implementation design. |
| Setting | 5 | Describe the setting, locations, and relevant dates, including periods of recruitment, exposure, follow-up, and data collection | 5 | - All ECEC centres providing long day care for children aged 0–5 years with a minimum 20 enrolled children and located in the Perth, Australia metropolitan area were eligible to take part. - Centre directors completed a survey in the period after tailoring their physical activity policy and prior to commencing implementation (pre-implementation period – January to June 2021). The post-intervention survey was collected from September 2021-March 2022 (post-implementation period). |
| Participants | 6 | (*a*) *Cohort study*—Give the eligibility criteria, and the sources and methods of selection of participants. Describe methods of follow-up  *Case-control study*—Give the eligibility criteria, and the sources and methods of case ascertainment and control selection. Give the rationale for the choice of cases and controls  *Cross-sectional study*—Give the eligibility criteria, and the sources and methods of selection of participants | 5 | - All ECEC centres providing long day care for children aged 0–5 years with a minimum 20 enrolled children and located in the Perth, Australia metropolitan area were eligible to take part. - Centre directors completed a survey in the period after tailoring their physical activity policy and prior to commencing implementation (pre-implementation period – January to June 2021). The post-intervention survey was collected from September 2021-March 2022 (post-implementation period). |
|  |  | (*b*) *Cohort study*—For matched studies, give matching criteria and number of exposed and unexposed  *Case-control study*—For matched studies, give matching criteria and the number of controls per case |  |  |
| Variables | 7 | Clearly define all outcomes, exposures, predictors, potential confounders, and effect modifiers. Give diagnostic criteria, if applicable | 5-6 | - Centre-level demographics were collected at pre-implementation. Director’s level of education was coded into two categories (lower: Secondary school/certificate/diploma vs. higher: University degree). Centre-level data was sourced from the Australian Children's Education and Care Quality Authority (ACECQA) website and included centre ratings for the Quality Rating 2: Children’s health and safety element: ‘Each child’s health and physical activity is supported and promoted’ (ratings: not assessed, working towards, meeting, exceeding) and number of approved places to determine centre size - small/medium, large, extra-large. Centre suburb postcode was used as a proxy for socio-economic status (low, middle, high) and derived from the Australian Bureau of Statistics’ Socio-Economic Indexes for Areas (SEIFA) - The 12-item Organisational Readiness for Implementing Change (ORIC) (35) measured ECEC centre change commitment and change efficacy at pre-implementation. - The Acceptability of Intervention Measure (AIM), Appropriateness of Intervention Measure (IAM) and Feasibility of Intervention Measure (FIM) scales, developed by Weiner and colleagues, (18) measured directors’ perceptions of the acceptability, appropriateness and feasibility of Play Active at pre-implementation. - Informed by the work of Proctor, et al., (17) a fidelity measure was constructed to measure overall implementation fidelity, adherence, dose, quality of delivery and participant responsiveness scores of Play Active at post-implementation. These were based on fidelity indictors set out in the Play Active protocol. |
| Data sources/ measurement | 8* | For each variable of interest, give sources of data and details of methods of assessment (measurement). Describe comparability of assessment methods if there is more than one group | 5-6 | Demographic measures   - Centre-level demographics were collected at pre-implementation. Director’s level of education was coded into two categories (lower: Secondary school/certificate/diploma vs. higher: University degree). Centre-level data was sourced from the Australian Children's Education and Care Quality Authority (ACECQA) website and included centre ratings for the Quality Rating 2: Children’s health and safety element: ‘Each child’s health and physical activity is supported and promoted’ (ratings: not assessed, working towards, meeting, exceeding) and number of approved places to determine centre size - small/medium, large, extra-large. Centre suburb postcode was used as a proxy for socio-economic status (low, middle, high) and derived from the Australian Bureau of Statistics’ Socio-Economic Indexes for Areas (SEIFA).   Organisational readiness   - The 12-item Organisational Readiness for Implementing Change (ORIC) measured ECEC centre change commitment and change efficacy at pre-implementation (see Supplementary Table 1: Organisational Readiness Measures). The ORIC has been shown to be valid, reliable and suitable for use at the organisational level (α= 0.85-0.94). Organisational capacity was measured at pre-implementation by the 5-item organisational capacity scale from the Program Sustainability Assessment Tool (PSAT). The PSAT tool is valid and reliable (organisational capacity α= 0.87). - The Acceptability of Intervention Measure (AIM), Appropriateness of Intervention Measure (IAM) and Feasibility of Intervention Measure (FIM) scales, developed by Weiner and colleagues, measured directors’ perceptions of the acceptability, appropriateness and feasibility of Play Active at pre-implementation (Supplementary Table 1). The AIM, IAM and FIM 4-item scales are valid and reliable (AIM α= 0.85, IAM α= 0.91, FIM α= 0.89) and designed to be customizable.   Fidelity measure   - Informed by the work of Proctor, et al., a fidelity measure was constructed to measure overall implementation fidelity, adherence, dose, quality of delivery and participant responsiveness scores of Play Active at post-implementation (Figure 1). These were based on fidelity indictors set out in the Play Active protocol (e.g., Personalize policy: centres select at least five from 25 practices within the physical activity policy template to focus on initially). Sources for fidelity indicator data included web analytics, selected educator evaluation survey questions and project administration records. The components of the fidelity measure items were converted to z-scores to produce individual scores for adherence, dose, quality of delivery and participant responsiveness (Supplementary Table 2) and then summed to produce an overall fidelity score. Full details of the development of the fidelity measures are in Supplementary Table 2. |
| Bias | 9 | Describe any efforts to address potential sources of bias | n/a |  |
| Study size | 10 | Explain how the study size was arrived at | n/a | Sample size was determined by the Play Active trial, not this study. Details available in the trial’s protocol paper. |

Continued on next page

| Quantitative variables | 11 | Explain how quantitative variables were handled in the analyses. If applicable, describe which groupings were chosen and why | 6 | Due to >95% of responses falling into the two highest of the five response options (disagree; somewhat disagree; neither agree nor disagree; somewhat agree; agree) for each of organisational change commitment, change efficacy, organisational capacity, acceptability, appropriateness and feasibility of intervention scales, responses were recoded into two categories (e.g., some or neutral commitment; committed) (Supplementary Table 1). |
| --- | --- | --- | --- | --- |
| Statistical methods | 12 | (*a*) Describe all statistical methods, including those used to control for confounding | 7-8 | Using SPSS v.28, descriptive statistics were calculated for all variables. One-way ANOVA was performed to analyze unadjusted associations between each independent variable (organisational commitment, efficacy and capacity; acceptability, appropriateness, feasibility) and the overall fidelity score. Subsequently, linear regression was used to model the six readiness to implement change variables separately with the overall fidelity score, after adjusting for ECEC centre socio-demographic characteristics, |
|  |  | (*b*) Describe any methods used to examine subgroups and interactions | n/a |  |
|  |  | (*c*) Explain how missing data were addressed | n/a |  |
|  |  | (*d*) *Cohort study*—If applicable, explain how loss to follow-up was addressed  *Case-control study*—If applicable, explain how matching of cases and controls was addressed  *Cross-sectional study*—If applicable, describe analytical methods taking account of sampling strategy | n/a |  |
|  |  | (*e*) Describe any sensitivity analyses | n/a |  |
| Results | | | | |
| Participants | 13* | (a) Report numbers of individuals at each stage of study—eg numbers potentially eligible, examined for eligibility, confirmed eligible, included in the study, completing follow-up, and analysed | Table 2 |  |
|  |  | (b) Give reasons for non-participation at each stage | n/a |  |
|  |  | (c) Consider use of a flow diagram | n/a |  |
| Descriptive data | 14* | (a) Give characteristics of study participants (eg demographic, clinical, social) and information on exposures and potential confounders | 8-9 | - Most ECEC intervention centres (73%) were large to very large with 58 or more approved places for children (Table 1). Around half of centres (48%) were in high socio-economic status (SES) areas and just over half (55%) were rated as ‘meeting the national quality standard’ for Quality Rating 2: Children’s health and safety which includes a focus on promoting children’s physical activity. (40) The majority of ECEC centre directors (80%) did not hold tertiary education qualifications. - The overall fidelity mean z-score for ECEC centres was 1.7 ± 5.0 (possible range: -7.97 - 12.33) at the 3-5-month follow up. |
|  |  | (b) Indicate number of participants with missing data for each variable of interest | Table 2 |  |
|  |  | (c) *Cohort study*—Summarise follow-up time (eg, average and total amount) |  | The overall fidelity mean z-score for ECEC centres was 1.7 ± 5.0 (possible range: -7.97 - 12.33) at the 3-5-month follow up. |
| Outcome data | 15* | *Cohort study*—Report numbers of outcome events or summary measures over time | Table 2 |  |
|  |  | *Case-control study—*Report numbers in each exposure category, or summary measures of exposure |  |  |
|  |  | *Cross-sectional study—*Report numbers of outcome events or summary measures |  |  |
| Main results | 16 | (*a*) Give unadjusted estimates and, if applicable, confounder-adjusted estimates and their precision (eg, 95% confidence interval). Make clear which confounders were adjusted for and why they were included | Table 3 & Supp Table 3 |  |
|  |  | (*b*) Report category boundaries when continuous variables were categorized | Table 3 |  |
|  |  | (*c*) If relevant, consider translating estimates of relative risk into absolute risk for a meaningful time period |  |  |

Continued on next page

| Other analyses | 17 | Report other analyses done—eg analyses of subgroups and interactions, and sensitivity analyses | n/a |  |
| --- | --- | --- | --- | --- |
| Discussion | | | | |
| Key results | 18 | Summarise key results with reference to study objectives | 13 | This study examined the association between ECEC centre organisational readiness and intervention acceptability, appropriateness and feasibility with the implementation fidelity of Play Active (an ECEC-specific physical activity policy intervention). Overall, implementation fidelity to Play Active was positively associated with factors of organisational readiness including organisational commitment, organisational capacity, intervention appropriateness and intervention acceptability. |
| Limitations | 19 | Discuss limitations of the study, taking into account sources of potential bias or imprecision. Discuss both direction and magnitude of any potential bias | 14 | The main Play Active trial was subject to limitations that impacted the current study, such as: a relatively short implementation period, self-selection of the ECEC sample and ECEC sector workforce challenges exacerbated by Covid-19 restrictions. Participating ECEC centres were metropolitan only, impacting the generalizability of our results for implementing Play Active in regional, rural or remote centres. In addition, the measures used in the current study were either adapted from those used in other settings or were adapted for the current study and therefore were not fully validated. There was little variation across some of the survey item responses resulting in response scales being dichotomized for data analysis. We were also unable to visit ECEC centres during the implementation period and relied on centre director self-reports rather than being able to objectively observe the implementation of Play Active in ECEC centres. Finally, as only long day care centres were included in the main study, these results may not be applicable to all types of ECEC centres (e.g., family day care, out of school care). |
| Interpretation | 20 | Give a cautious overall interpretation of results considering objectives, limitations, multiplicity of analyses, results from similar studies, and other relevant evidence |  | This study explored the relationships between organisational readiness and implementation fidelity of the ECEC-based Play Active physical activity policy intervention. Our results demonstrated that increases in Play Active implementation fidelity were significantly associated with higher levels of organisational commitment, organisational capacity for change, intervention acceptability and appropriateness. These results are important for informing the future implementation and sustainability of physical activity interventions in the ECEC setting as many rarely make significant or lasting increases in children’s activity levels. In order to support successful implementation and the subsequent positive impact on child physical activity levels and health, further research should be undertaken to determine how best to incorporate organisational readiness strategies into future ECEC-specific physical activity policy interventions |
| Generalisability | 21 | Discuss the generalisability (external validity) of the study results |  | Participating ECEC centres were metropolitan only, impacting the generalizability of our results for implementing Play Active in regional, rural or remote centres. |
| Other information | |  | | |
| Funding | 22 | Give the source of funding and the role of the funders for the present study and, if applicable, for the original study on which the present article is based |  | Funding:  This work is partially supported through the Australian Research Council's Centre of Excellence for Children and Families over the Life Course (#CE200100025). EW is supported by an Australian Government Research Training Program (RTP) Stipend and RTP Fee-Offset Scholarship through The University of Western Australia and a Minderoo Foundation Top-up Scholarship through the Telethon Kids Institute. NP is supported by the Australian Government through the Australian Research Council’s Centre of Excellence for Children and Families over the Life Course [number CE200100025]. HC is supported by a National Heart Foundation Future Leader Fellowship (#102549). None of the funding bodies had a role in study design, data collection, analysis, report writing or publication of this article. |

*Give information separately for cases and controls in case-control studies and, if applicable, for exposed and unexposed groups in cohort and cross-sectional studies.

**Note:** An Explanation and Elaboration article discusses each checklist item and gives methodological background and published examples of transparent reporting. The STROBE checklist is best used in conjunction with this article (freely available on the Web sites of PLoS Medicine at http://www.plosmedicine.org/, Annals of Internal Medicine at http://www.annals.org/, and Epidemiology at http://www.epidem.com/). Information on the STROBE Initiative is available at www.strobe-statement.org.
