# Supplementary material for: Organizational readiness and implementation fidelity of an early childhood education and care-specific physical activity policy intervention: findings from the Play Active trial
Source: J Public Health (Oxf). 2023 Nov 22;46(1):158–67. doi: 10.1093/pubmed/fdad221 (PMC10901271; doi:10.1093/pubmed/fdad221)
Supplement: Supplementary_materials_fdad221 [file supplementary_materials_fdad221.zip › Supplementary_materials_fdad221/Supplemental Figures 1a & 1b.docx]

**Supplemental Figure 1a. Play Active ECEC services meeting fidelity criteria by demographic variables – Adherence and Dose**

**
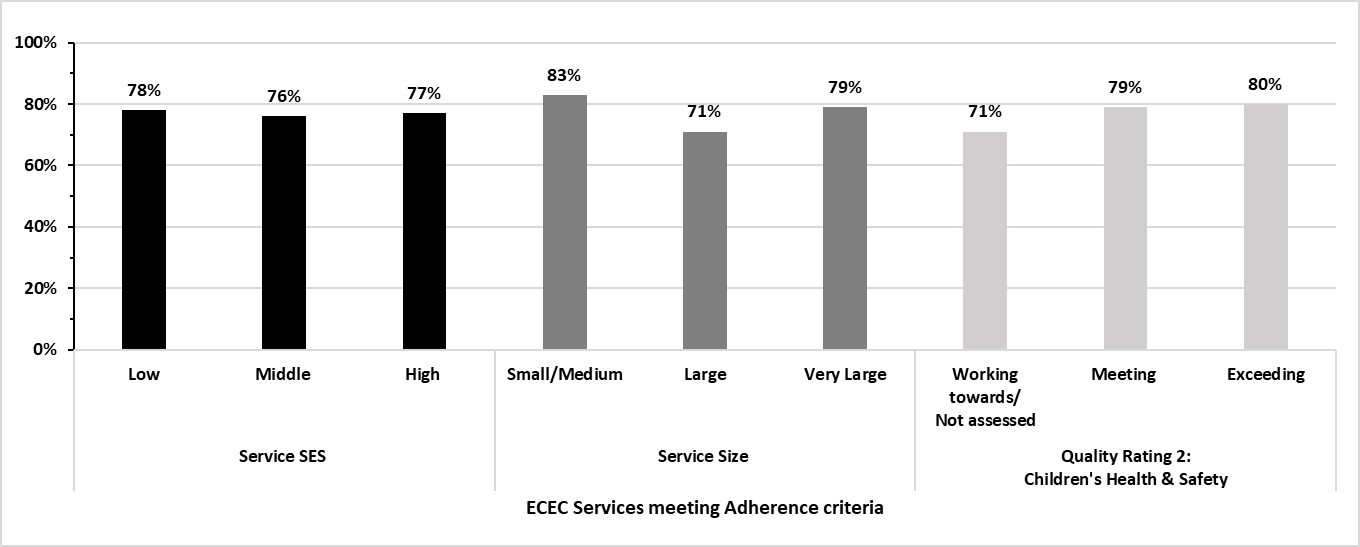
**

**
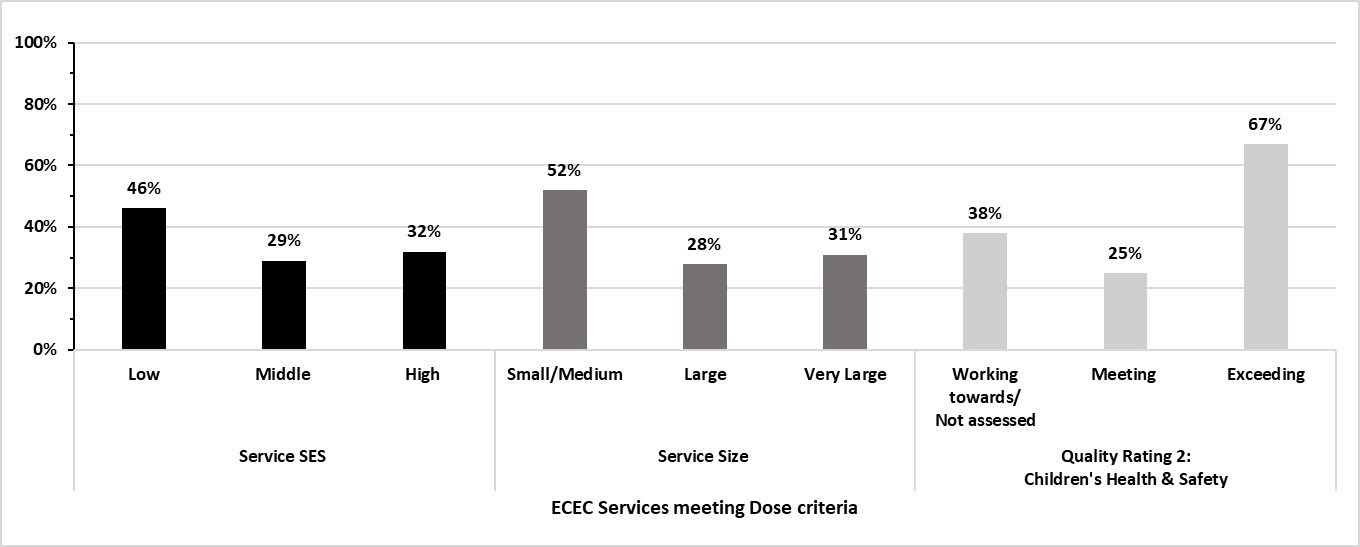
**

*****

**Supplemental Figure 1b. Play Active ECEC services meeting fidelity criteria by demographic variables – Quality of Delivery and Participant Responsiveness.**

**
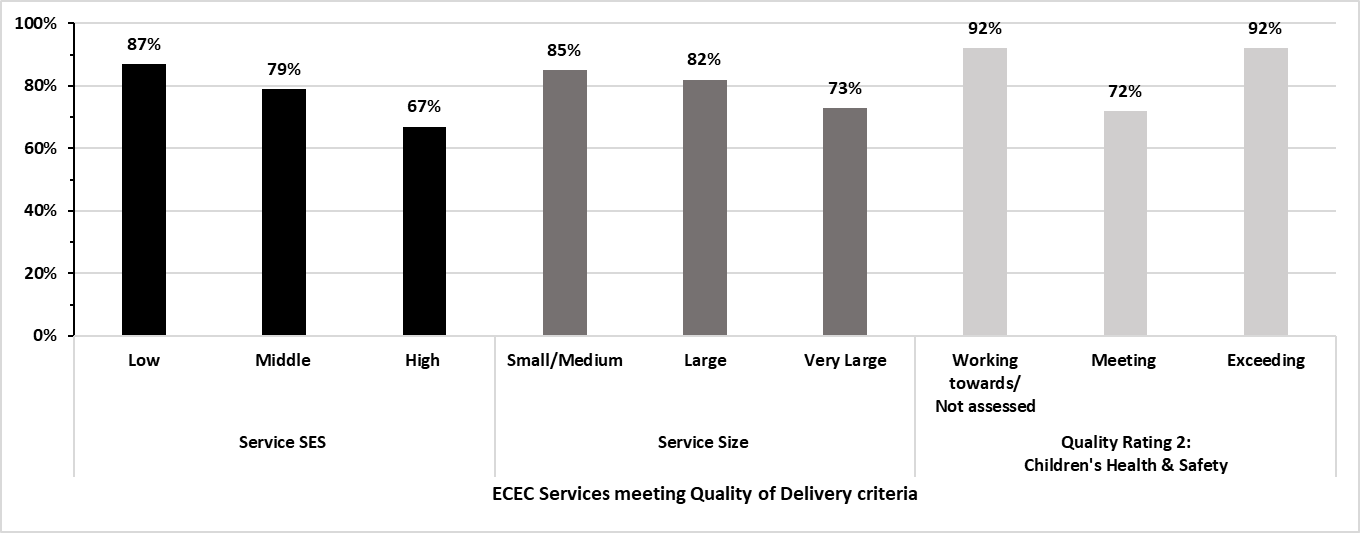
**

**
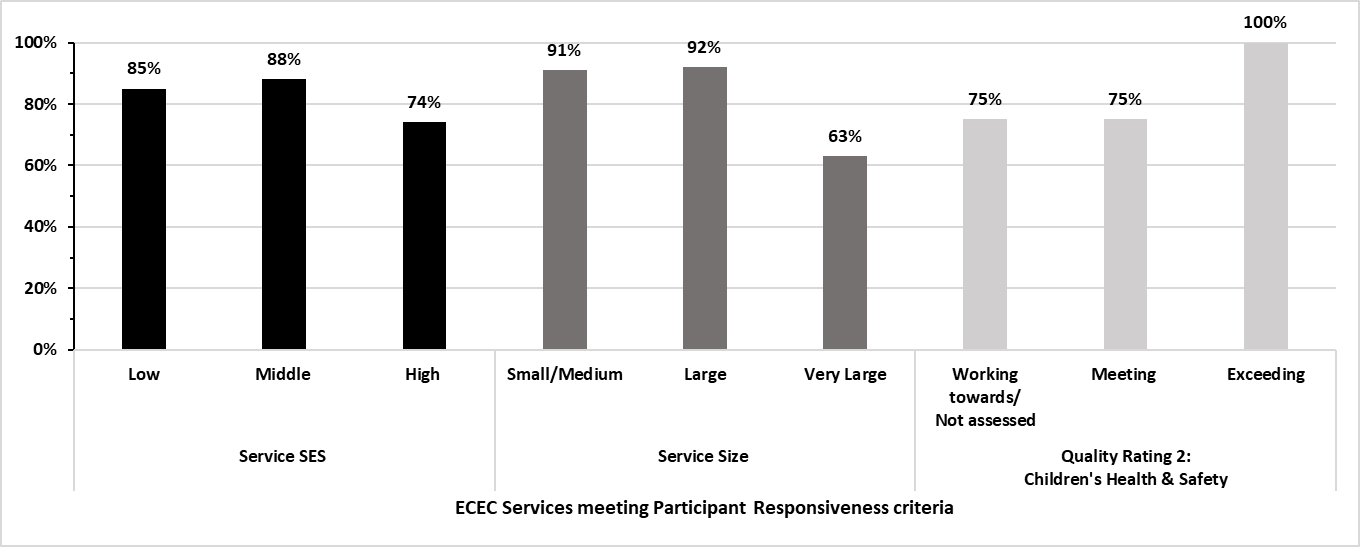
**

*****

* p<0.05
