# Supplementary material for: Organizational readiness and implementation fidelity of an early childhood education and care-specific physical activity policy intervention: findings from the Play Active trial
Source: J Public Health (Oxf). 2023 Nov 22;46(1):158–67. doi: 10.1093/pubmed/fdad221 (PMC10901271; doi:10.1093/pubmed/fdad221)
Supplement: Supplementary_materials_fdad221 [file supplementary_materials_fdad221.zip › Supplementary_materials_fdad221/Supplementary Table 1.docx]

**Supplementary Table 1: Organisational Readiness Measures**

| **Scale** | **Items** | **Scoring** |
| --- | --- | --- |
| Adapted ORIC change efficacy scale ^39^ | Educators who work here feel confident that our service is invested in implementing our Physical Activity Policy. | 5-pt Likert:   1. Disagree 2. Somewhat disagree 3. Neither agree nor disagree 4. Somewhat agree 5. Agree   Recoded scale score for analysis:   1. Some or neutral efficacy (1 - 4, above, recoded) 2. Efficacious (5, above, recoded) |
|  | Educators who work here are committed to implementing our Physical Activity Policy. |  |
|  | Educators who work here will do whatever it takes to implement our Physical Activity Policy. |  |
|  | Educators who work here want to implement our Physical Activity Policy. |  |
|  | Educators who work here feel confident that they can keep the momentum going in implementing our Physical Activity Policy. |  |
|  | Educators who work here are determined to implement our Physical Activity Policy. |  |
|  | Educators who work here are motivated to implement our Physical Activity Policy. |  |
| Adapted ORIC change commitment scale ^39^ | Educators who work here feel confident that they can keep track of progress in implementing our Physical Activity Policy. | 5-pt Likert:   1. Disagree 2. Somewhat disagree 3. Neither agree nor disagree 4. Somewhat agree 5. Agree   Recoded scale score for analysis:   1. Some or neutral commitment (1 - 4, above, recoded) 2. Committed (5, above, recoded) |
|  | Educators who work here feel confident that this service can support people as they implement our Physical Activity Policy. |  |
|  | Educators who work here feel confident that they can handle the challenges that might arise in implementing our Physical Activity Policy. |  |
|  | Educators who work here feel confident that they can coordinate tasks so that our Physical Activity Policy implementation goes smoothly. |  |
|  | Educators who work here feel confident that they can manage the politics of implementing our Physical Activity Policy. |  |

| **Scale** | **Items** | **Scoring** |
| --- | --- | --- |
| Adapted PSAT organisational capacity scale ^42^ | Our Physical Activity Policy will be well integrated into the practices of this service. | 5-pt Likert:   1. No extent 2. Little extent 3. Some extent 4. Great extent 5. Very great extent   Recoded scale score for analysis:   1. No/little/some or great extent 2. Very great extent |
|  | Organisational systems are in place to support the various Physical Activity Policy recommendations. |  |
|  | Our leaders will effectively voice the vision of our Physical Activity Policy to our parents and families. |  |
|  | Our leaders efficiently manage staffing and service resources. |  |
|  | This service has adequate educators to achieve our Physical Activity Policy's goals. |  |
| Customized AIM measure ^24^ | Our Physical Activity Policy meets educators' approval. | 5-pt Likert:   1. Disagree 2. Somewhat disagree 3. Neither agree nor disagree 4. Somewhat agree 5. Agree   Recoded for analysis:   1. Somewhat acceptable (1 - 4, above, recoded) 2. Acceptable (5, above, recoded) |
|  | Our Physical Activity Policy is appealing to educators. |  |
|  | Educators like our Physical Activity Policy. |  |
|  | Educators welcome our Physical Activity Policy. |  |
| Customized IAM measure ^24^ | Our Physical Activity Policy seems fitting. | 5-pt Likert:   1. Disagree 2. Somewhat disagree 3. Neither agree nor disagree 4. Somewhat agree 5. Agree   Recoded for analysis:   1. Somewhat appropriate (1 - 4, above, recoded) 2. Appropriate (5, above, recoded) |
|  | Our Physical Activity Policy seems suitable. |  |
|  | Our Physical Activity Policy seems applicable. |  |
|  | Our Physical Activity Policy seems like a good match. |  |

| Customed FIM measure ^24^ | Our Physical Activity Policy seems implementable. | 5-pt Likert:   1. Disagree 2. Somewhat disagree 3. Neither agree nor disagree 4. Somewhat agree 5. Agree   Recoded for analysis:   1. Somewhat feasible (1 - 4, above, recoded) 2. Feasible (5, above, recoded) |
| --- | --- | --- |
|  | Our Physical Activity Policy seems possible. |  |
|  | Our Physical Activity Policy seems do-able. |  |
|  | Our Physical Activity Policy seems easy to use |  |
