# Supplementary material for: Organizational readiness and implementation fidelity of an early childhood education and care-specific physical activity policy intervention: findings from the Play Active trial
Source: J Public Health (Oxf). 2023 Nov 22;46(1):158–67. doi: 10.1093/pubmed/fdad221 (PMC10901271; doi:10.1093/pubmed/fdad221)
Supplement: Supplementary_materials_fdad221 [file supplementary_materials_fdad221.zip › Supplementary_materials_fdad221/Supplementary Table 2.docx]

**Supplementary Table 2: Fidelity measure**

**Adherence Fidelity**

| 1 | **IMPLEMENTATION STEP 1**  **Personalise policy:** ECEC service staff will be encouraged to personalise the physical activity policy template to suit their service and the needs of their attending children and families. Services are asked to select at least five from 25 practices to prioritise during implementation.  To support ECEC services in this, seven high impact and low effort practices, based on previous testing ^51^ are indicated. | **Scoring**  All practices  selected at least 5/25 = 1  selected less than 5/25 = 0  Key practices  Selected 5 or more = 5  Selected 4 = 4  Selected 3 = 3  Selected 2 = 2  Selected 1 = 1  Selected 0 = 0 | **Data Source**   - Play Active policy template   **Timepoint:** Pre-implementation   - Play Active policy template   **Timepoint:** Pre-implementation |
| --- | --- | --- | --- |
| 2 | **IMPLEMENTATION STEP 2**  **Policy review and approval:** Services are asked to submit their Play Active physical activity policy (based on the template) to the research team for approval. Two Play Active Project Officers independently reviewed and approved policies that meet met minimum criteria. The minimum requirements for physical activity policies to be approved are: two (out of two) key statements; nine (out of nine) recommendations; and at least five (out of 25) practices (Imp Step 1) to prioritise during implementation. | **Scoring**  Policy returned and approval  0 = Not returned  0 = Returned and not approved  1 = Returned and approved | **Data Source**   - Play Active policy template   **Timepoint:** Pre implementation |
| 3 | **IMPLEMENTATION STEP 3**  **Resource guide:** An evidence-informed resource guide (hard copy and digital PDF) with practical tips describing how to implement each of the 25 physical activity practices into daily practice has been developed. The 25 practices are consistent with the policy template and outline practical strategies educators can use, an evidence-informed explanation of what it means, and various evidence-based helpful resources for more information. One physical copy of the resource guide is provided to each service as well as an electronic copy. | **Scoring**  *Q: Did you use the Play Active Resource Guide?*  0 = Not used  1 = Used | **Data Source**   - Educator survey - aggregated to service level   **Timepoint:** Post-implementation |
| 4 | **IMPLEMENTATION STEP 4**  **Brief assessment tool:** A brief monitoring tool has been created for educators to monitor children’s physically activity whilst attending ECEC. The Energetic Play Assessment Tool (EPAT) was adapted from a brief instrument developed by Rice and colleagues ^52^. For each child, educators record how much of a typical day in the last month was spent energetically playing (e.g., running, jumping, skipping, dancing, riding, climbing and energetic games) indoors and outdoors. The tool is included as part of the Play Active resource guide. | **Scoring**  EPAT data collection (max. score 2)  2 = Data collected pre and post  1 = Data collected pre or post  0 = No data collected | **Data Source:**   - Project administration records - aggregated to service level   **Timepoint**: Pre-implementation; Post-implementation |
| 5 | **IMPLEMENTATION STEP 6a**  **Project Officer implementation support:** including both weekly follow-up (phone and/or email) to complete the policy review and a mid-implementation prompt (phone call) to determine whether policy implementation has commenced. Service directors were provided weekly email and phone call reminders to return their policy for review for up to 2 months.  Mid-implementation check-up (approx.6 weeks from pre-implementation) | **Scoring**  No. of contacts for policy return (max. score 5):  1 = 4 or more contacts  2 = 3 contacts  3 = 2 contacts  4 = 1 contact  5 = 0 contacts  *Q: Started implementing procedures?*  0 = No  1 = Yes | **Data Source**   - Project administration records – contact count   **Timepoint:** Post-implementation   - Mid-implementation telephone contact   **Timepoint:** Mid-implementation |

**Dose Fidelity:**

| 1 | **IMPLEMENTATION STEP 5**  **Professional development**: Online training was available to upskill educators in providing more physical activity opportunities for children in their care, including specific skills on developing fundamental movement skills and active play-based learning. | **Scoring**  PD amount accessed by service (max. score 2):  0 = <25% staff started but no full completion  1 = <25% staff completed or >25% partial completion  2 = >25% staff completed | **Data Source**   - Professional development web analytics   **Timepoint:** Post-implementation |
| --- | --- | --- | --- |
| 2 | **IMPLEMENTATION STEP 6b**  **Project Officer implementation support:** Mid-implementation prompt (phone call) at approx. 6 weeks into implementation period to determine whether use of implementation supports had commenced | **Scoring**  *Q: Have you been using the Play Active resource guide?*  0 = No  1 = Yes  *Q: Have you accessed the professional development?*  0 = No  1 = Yes | **Data Source**   - Mid-implementation telephone contact   **Timepoint:** Mid-implementation |

**Quality of Delivery** **Fidelity:**

| 1 | **To measure organisational-level perceptions of quality of delivery of Play Active in individual services.**  Selected from the Implementation Leadership Scale (ILS) & adapted from Proactive, Supportive and Perseverant scales ^42^ (adapted to context) | **Scoring**  *Q: To what extent did the following occur in your service:*   1. *Our service developed a plan to facilitate implementation of our Physical Activity Policy.* 2. *Our service reacted to issues regarding the implementation of our Physical Activity Policy by openly and effectively addressing the problem(s).* 3. *Our service recognizes and appreciates educators’ efforts toward successful implementation of our Physical Activity Policy.*   0 = Not at all  1 = Slight extent  2 = Moderate extent  3 = Great extent  4 = Very great extent | **Data Source**   - Director survey   **Timepoint:** Post-implementation |
| --- | --- | --- | --- |

| 2 | **To measure Play Active dissemination from Director to Educators in individual services.**  Context-specific to Play Active  New item – no psychometrics available | **Potential scoring**  *Q: Which of the following were used:*   1. *While leading into implementing your physical activity policy in your service?* 2. *While implementing your physical activity policy in your service?*  - Email messages - Staff noticeboard - Staff meeting - Staff ‘champion’ - Printed information - Professional development in children’s physical activity (other than that provided by Play Active) - Own physical activity resources - Other | **Data Source**   - Director survey - sum score for each condition   **Timepoint:** Post-implementation |
| --- | --- | --- | --- |

**Participant responsiveness**

| 1 | **To measure organisational-level perception of staff engagement and response to Play Active.**  Based on the work of Carroll, et al. ^53^  New items - no psychometrics available | **Scoring**  *Q: Thinking about the implementation of your Physical Activity Policy, please rate how much you agree or disagree with the following statements.*   1. Educators think the Play Active Program is useful for increasing children’s physical activity. 2. Educators are willing to engage in the Play Active Program. 3. Educators understand the physical activity recommendations in our Physical Activity Policy. 4. Educators are confident to apply the physical activity recommendations in our Physical Activity Policy. 5. Educators are enthusiastic about the Play Active Program.   0 = Strongly disagree  1 = Disagree  2 = Neither agree nor disagree  3 = Agree  4 = Strongly agree | **Data Source**   - Director survey - sum score for each condition   **Timepoint:** Post-implementation |
| --- | --- | --- | --- |
