# Supplementary material for: Organizational readiness and implementation fidelity of an early childhood education and care-specific physical activity policy intervention: findings from the Play Active trial
Source: J Public Health (Oxf). 2023 Nov 22;46(1):158–67. doi: 10.1093/pubmed/fdad221 (PMC10901271; doi:10.1093/pubmed/fdad221)
Supplement: Supplementary_materials_fdad221 [file supplementary_materials_fdad221.zip › Supplementary_materials_fdad221/Supplementary Table 3.docx]

**Supplementary Table 3. Unadjusted associations between readiness variables and fidelity to Play Active**

| Scale variable ^a^ |  | (n=36) | |
| --- | --- | --- | --- |
|  |  | MD (95%CI) | p |
| Organisational commitment  *(Committed - Some or neutral commitment)* |  | 5.26 (2.22, 8.31) | 0.001* |
|  |  |  |  |
| Organisational efficacy  *(Efficacious - Some or neutral efficacy)* |  | 4.93 (1.47, 8.40) | 0.007* |
|  |  |  |  |
| Organisational capacity  *(Very great extent - Some or great extent)* |  | 3.79 (0.27, 7.31) | 0.036* |
|  |  |  |  |
| Acceptability of intervention  *(Acceptable - Somewhat acceptable or neutral)* |  | 5.32 (2.33, 8.30) | <0.001* |
|  |  |  |  |
| Appropriateness of intervention  *(Appropriate - Somewhat appropriate or neutral)* |  | 5.57 (3.15, 7.99) | <0.001* |
|  |  |  |  |
| Feasibility of intervention  *(Feasible - Somewhat feasible or neutral)* |  | 2.54 (-1.65, 6.73) | 0.196 |

*p<0.05

^a^ Groups compared using independent t-tests
